# Supplementary material for: MicroRNA cluster miR-17-92 regulates multiple functionally related voltage-gated potassium channels in chronic neuropathic pain
Source: Nat Commun. 2017 Jul 5;8:16079. doi: 10.1038/ncomms16079 (PMC5504285; doi:10.1038/ncomms16079)
Supplement: Supplementary Information [file ncomms16079-s1.pdf]

File name: Supplementary Information
Description: Supplementary Figures and Supplementary Tables

File name: Supplementary Data 1
Description: Downregulated putative target genes of miR-18a, miR-19a/b and miR-92a

File name: Supplementary Data 2
Description: Significantly-changed genes that showed fold change of >1.5

File name: Peer Review File
Description:

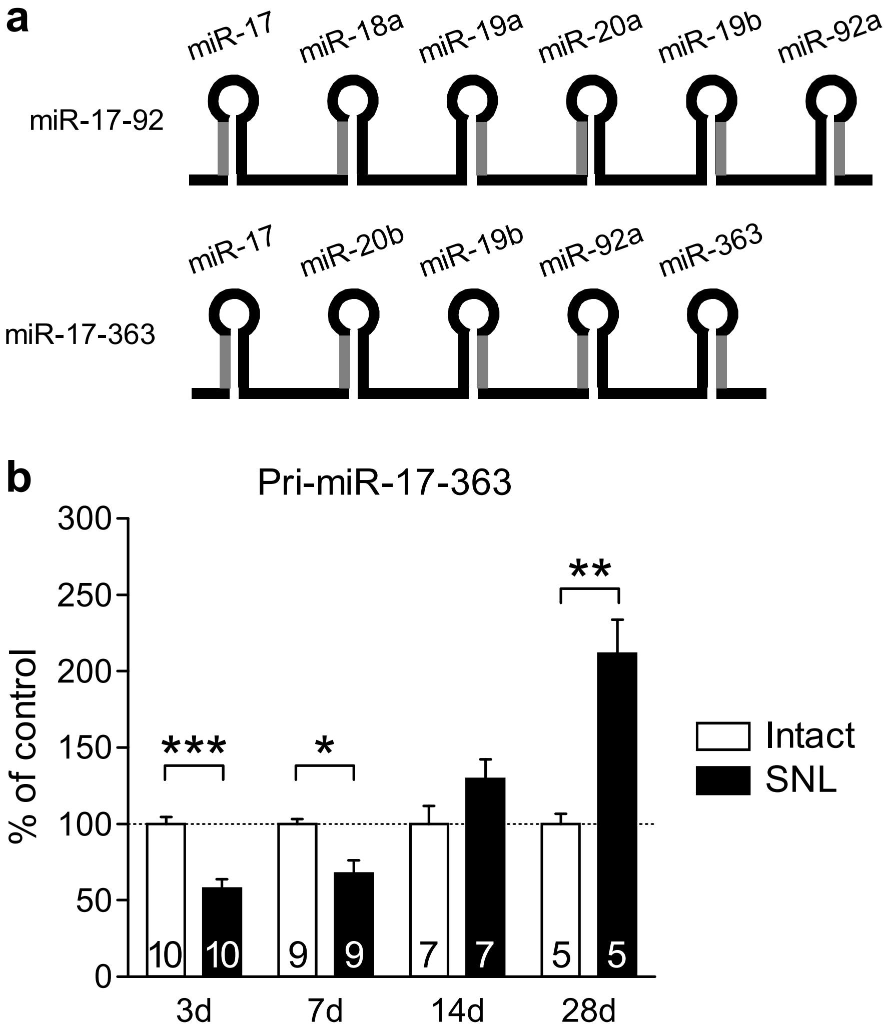


**Supplementary Figure 1 miR-17-92 paralog expression is not consistently changed after nerve injury.** (**a**) Schematic representation of miR-17-92 and its paralog miR-17-363 primary transcripts. These transcripts encode several similar mature miRNAs. (**b**) Expression of miR-17-363 primary transcript in the L5 DRG over time after SNL. Numbers of samples are shown at the base of each bar. Error bars are s.e.m.. **P* <0.05, ***P* <0.01 and ****P* <0.001 compared with the contralateral intact side, paired *t*-test.

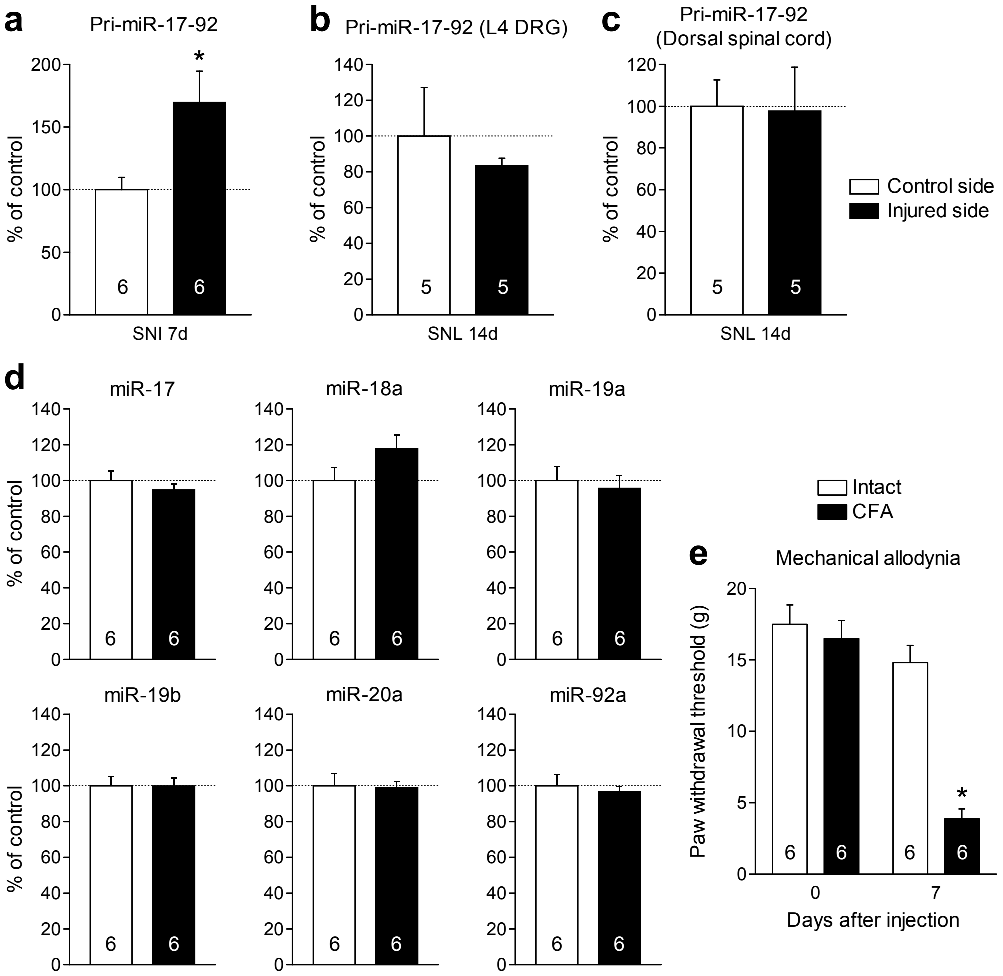


**Supplementary Figure 2 miR-17-92 expression is not influenced by inflammatory pain.** (**a**–**c**) Expression of pri-miR-17-92 in the L4 DRG 7 days after spared nerve injury (SNI) (**a**) and in the injury-spared L4 DRG (**b**) and L5 dorsal spinal cord (**c**) 14 days after SNL. **P* <0.05 compared with the contralateral intact side, paired *t*-test. (**d**) Expression of mature miR-17-92 cluster miRNAs in the L5 DRG 7 days after administration of intraplantar complete Freund's adjuvant (CFA). (**e**) Paw withdrawal responses to mechanical stimuli evaluated at day 7 on the CFA-injected and contralateral sides. Numbers of samples or animals are indicated at the base of each bar. Error bars are s.e.m.. **P* <0.05 compared with the contralateral intact side, Mann–Whitney *U* test.

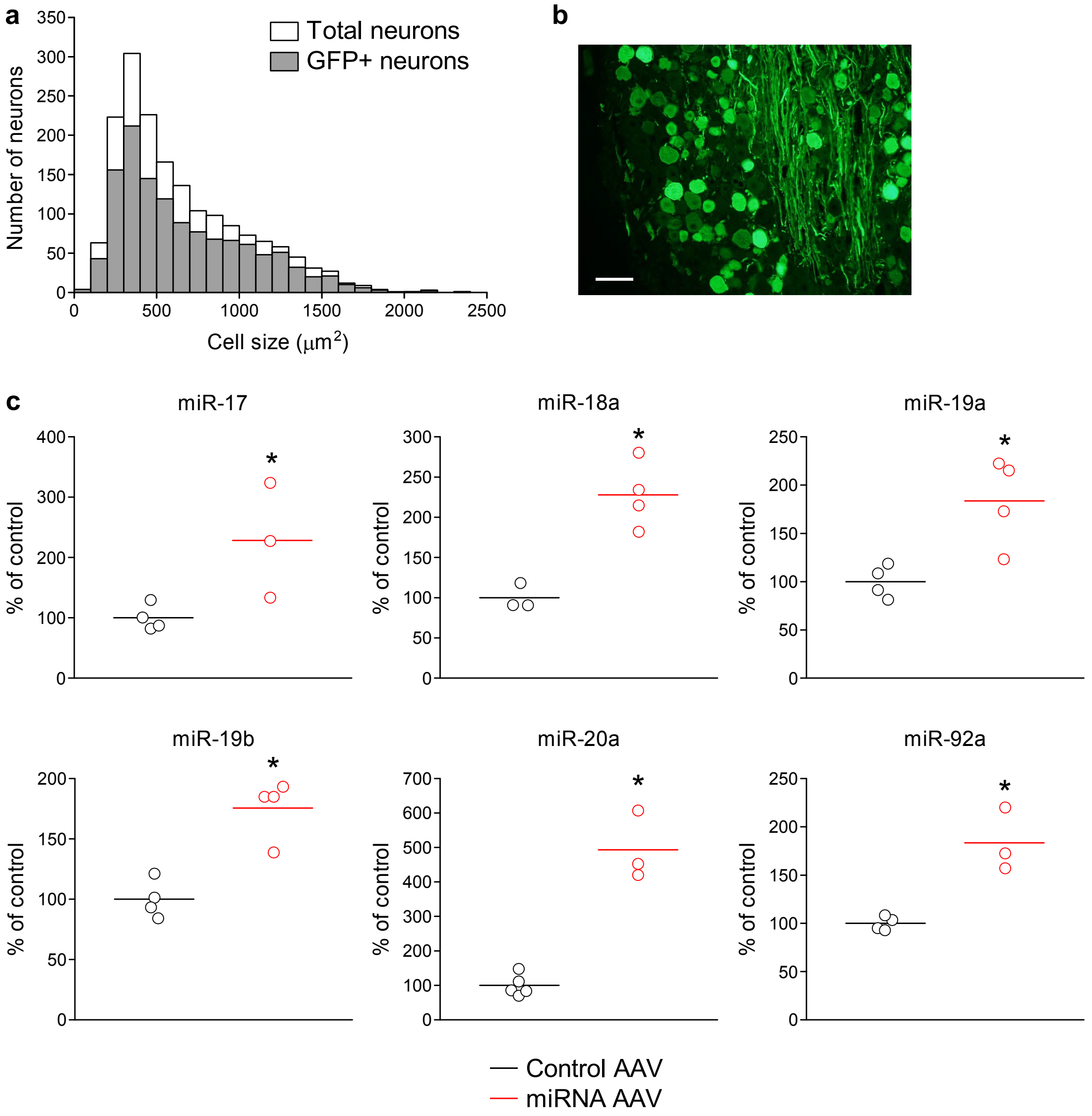


**Supplementary Figure 3 AAV vector encoding each miR-17-92 cluster member increases corresponding miRNA expression level.** (**a**) Size distribution of GFP-positive neurons 7 days after AAV vector administration. Two DRG sections obtained from individual rats were counted (total 3 rats). (**b**) Representative image of EGFP immunofluorescence in the L5 DRG 7 days after injection of the control AAV vector encoding EGFP. Scale bar, 100 μm. (**c**) Expression of miR-17-92 cluster members in the L5 DRG 7 days after the control or miR-17-92 AAV vector administration. *$P$ <0.05, Mann-Whitney $U$ test.

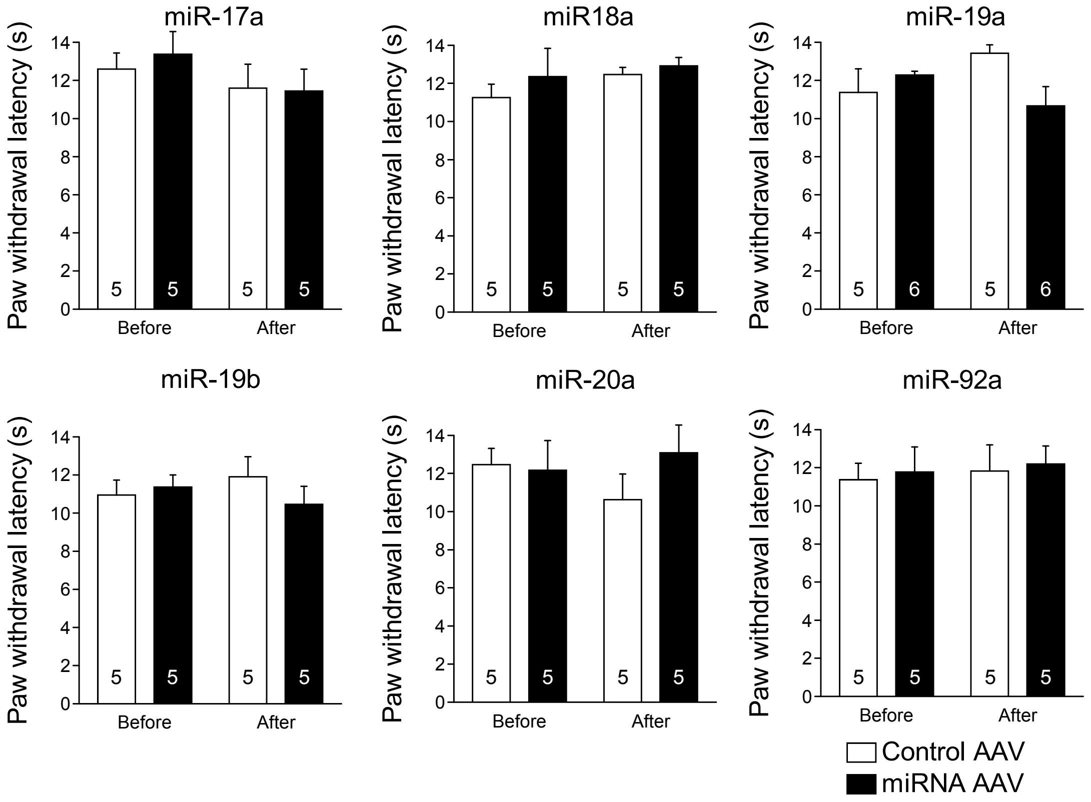


**Supplementary Figure 4 Thermal sensitivity is not affected by overexpression of each miR-17-92 cluster member.** Paw withdrawal latencies to thermal stimuli were evaluated on the injected sides before and 7 days after administration of AAV vector encoding an individual miR-17-92 cluster member. Numbers of animals are indicated at the base of each bar. Error bars are s.e.m..

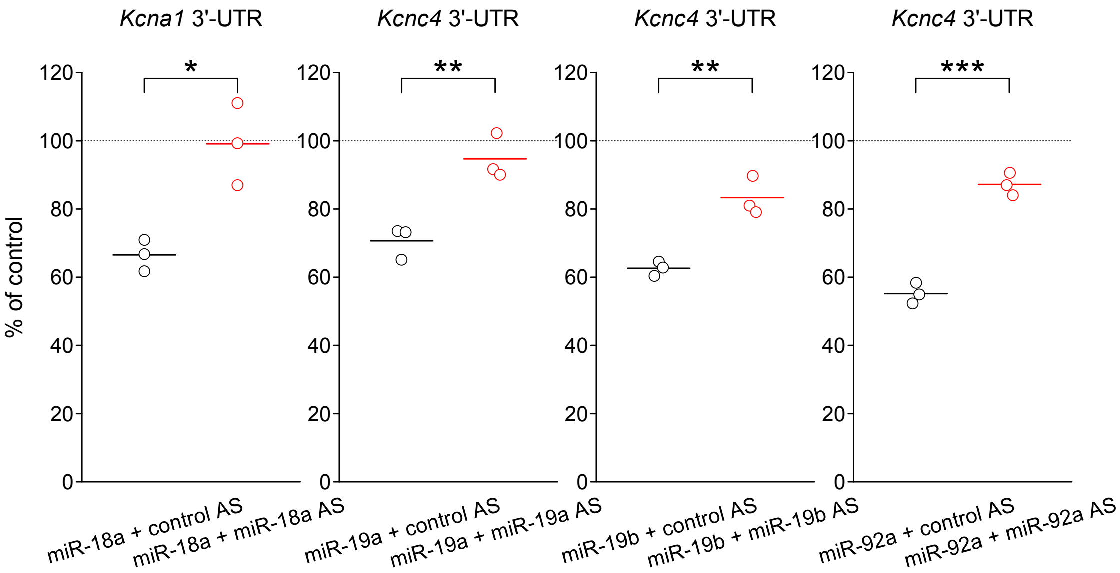


**Supplementary Figure 5 TuD antisense RNA against miRNA prevents miRNA-mediated inhibition of luciferase activity.** Activity of luciferase with the *Kcna1* or *Kcnc4* 3′-UTR in HEK293 cells transfected with plasmid vector expressing each miR-17-92 cluster miRNA. HEK293 cells were pre-transfected with control antisense (AS) RNA or anti-miRNA AS RNA. **P* <0.05, ***P* <0.01 and ****P* <0.001, unpaired *t*-test.

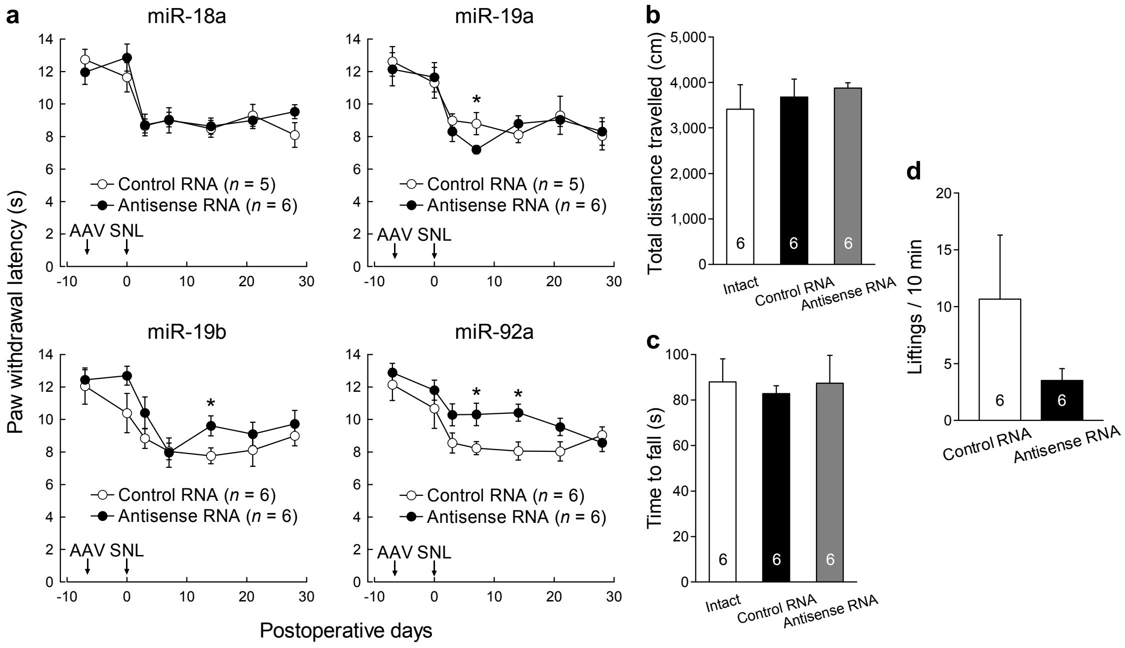


**Supplementary Figure 6 Thermal hyperalgesia, motor function and spontaneous pain are not affected by blockade of miR-17-92.** (**a**) Paw withdrawal latencies to thermal stimuli were evaluated on the AAV-injected/SNL side. AAV vector encoding the control TuD antisense RNA or TuD antisense RNA against each miR-17-92 cluster member was administered to the L5 DRG 7 days before SNL (indicated by arrows). $^*P < 0.05$, unpaired *t*-test. (**b,c**) Open field test (**b**) and rotarod test (**c**) were performed in intact rats and rats injected with AAV vector encoding control TuD antisense RNA or mixture of AAV vectors encoding TuD antisense RNAs against miR-18a, miR-19a, miR-19b and miR-92a 6 days (**b**) and 7 days (**c**) after injection, respectively. (**d**) Combined neuropathic and inflammatory pain model was produced 7 days after injection of AAV vector encoding control TuD antisense RNA or mixture of AAV vectors encoding TuD antisense RNAs against miR-18a, miR-19a, miR-19b and miR-92a. Spontaneous paw liftings were counted for 10 min at day 7 after SNL + CFA injection. Numbers of animals are indicated at the base of each bar. Error bars are s.e.m..

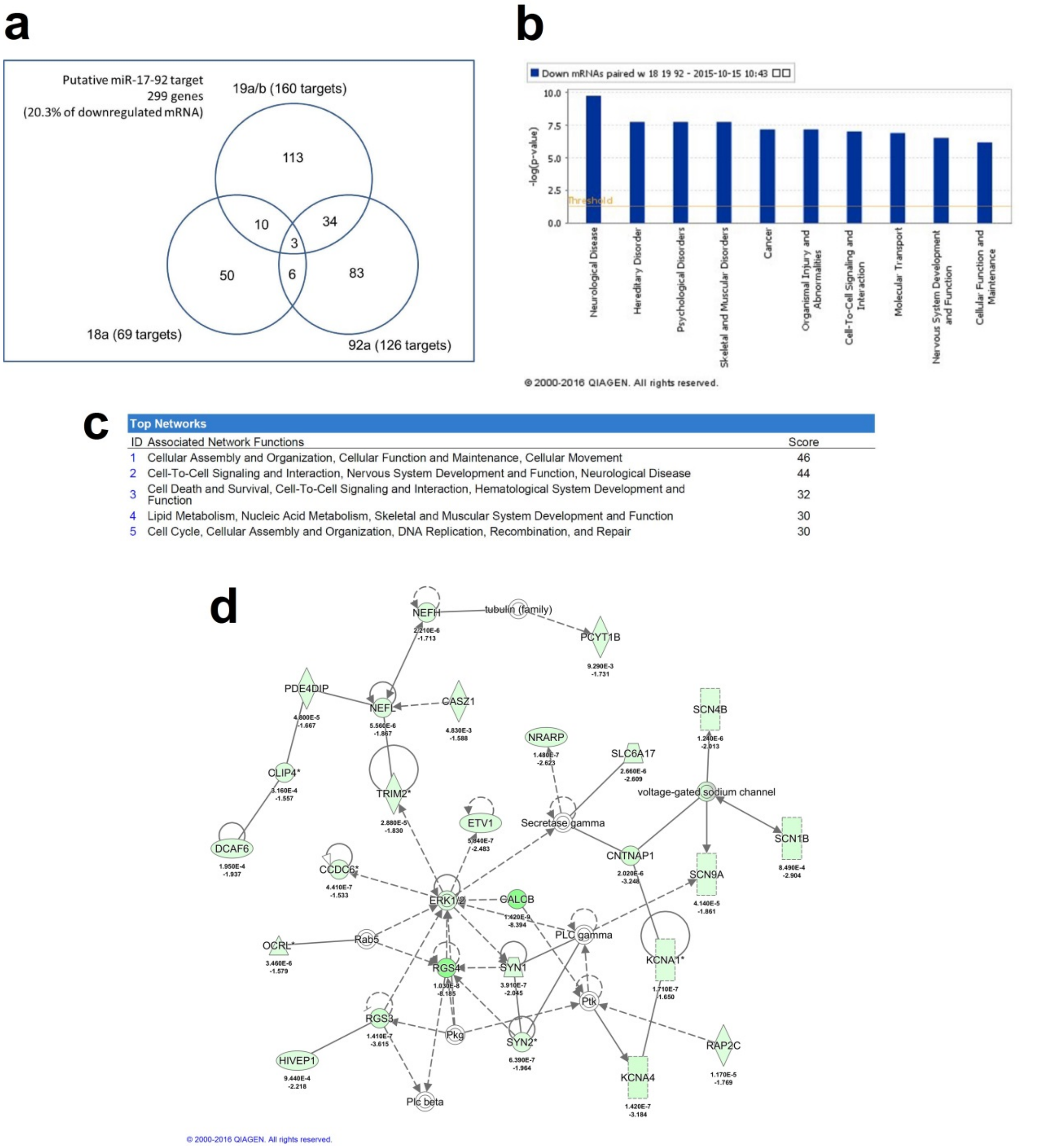


| ID | Associated Network Functions | Score |
|---|---|---|
| 1 | Cellular Assembly and Organization, Cellular Function and Maintenance, Cellular Movement | 46 |
| 2 | Cell-To-Cell Signaling and Interaction, Nervous System Development and Function, Neurological Disease | 44 |
| 3 | Cell Death and Survival, Cell-To-Cell Signaling and Interaction, Hematological System Development and Function | 32 |
| 4 | Lipid Metabolism, Nucleic Acid Metabolism, Skeletal and Muscular System Development and Function | 30 |
| 5 | Cell Cycle, Cellular Assembly and Organization, DNA Replication, Recombination, and Repair | 30 |

**Supplementary Figure 7 Bioinformatic analysis of target genes for pain-relevant miR-17-92 cluster members using IPA.** (**a**) Venn diagram of predicted targets for each miRNA. Top 10 downstream biological processes (**b**) and top five network functions (**c**) associated with downregulation of the predicted target genes. (**d**) Molecular network formed by predicted target genes (green) associated with the network of cell-to-cell signaling and interaction, nervous system development and function, and neurological disease.

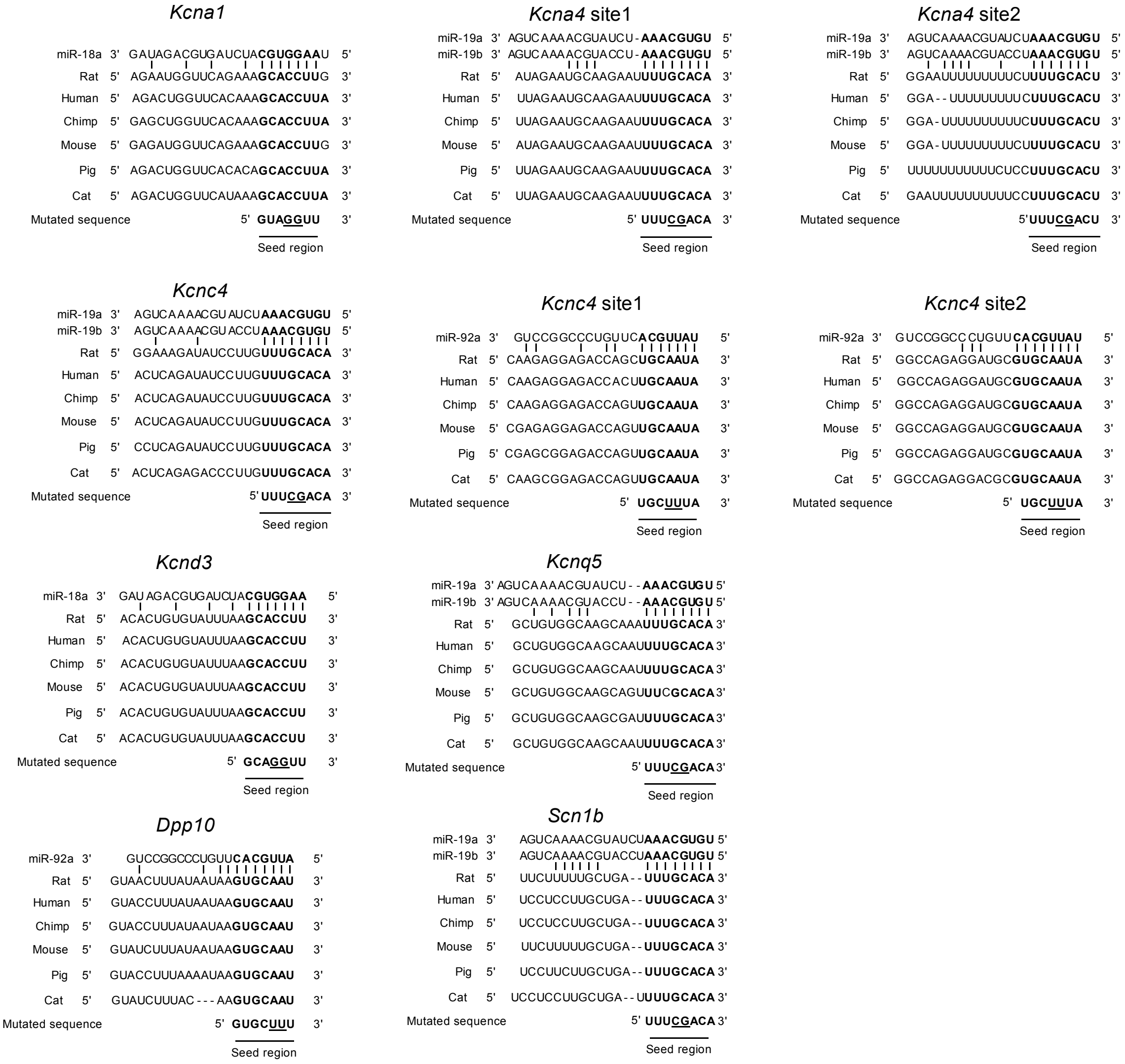


**Supplementary Figure 8 Schematic representation of miR-17-92 cluster member sequences and their target sequences within potassium channel subunit 3′-UTRs.** The seed sequences are indicated in bold. Target sequences are well conserved among mammals.

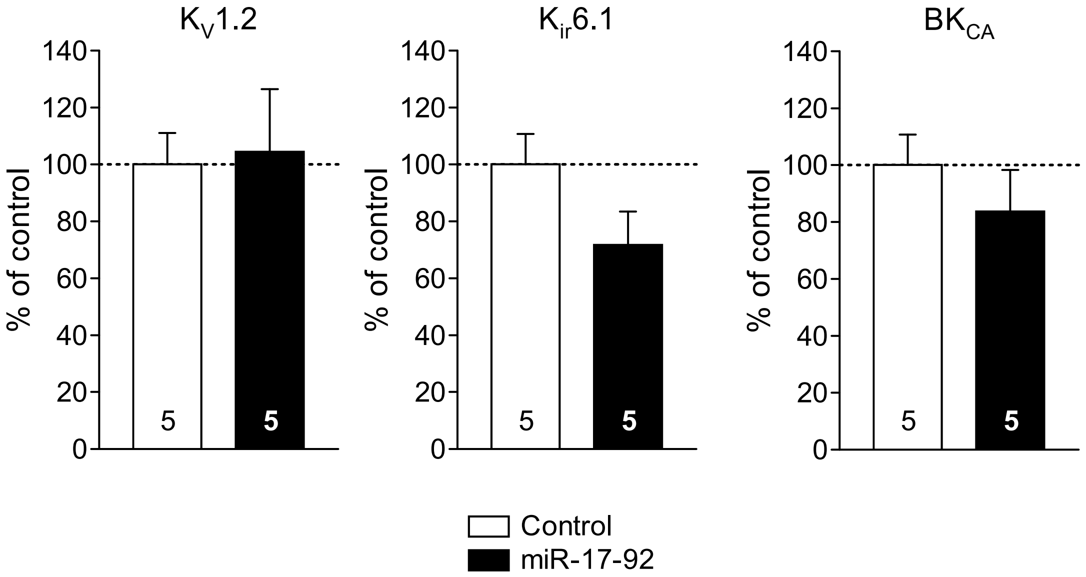


**Supplementary Figure 9 Expression of pain-related potassium channels that were not predicted as miR-17-92 targets.** Expressions of potassium channel subunit mRNAs were examined in the L5 DRG 7 days after control or miR-17-92 AAV vector administration. Numbers of samples are indicated at the base of each bar. Error bars are s.e.m..

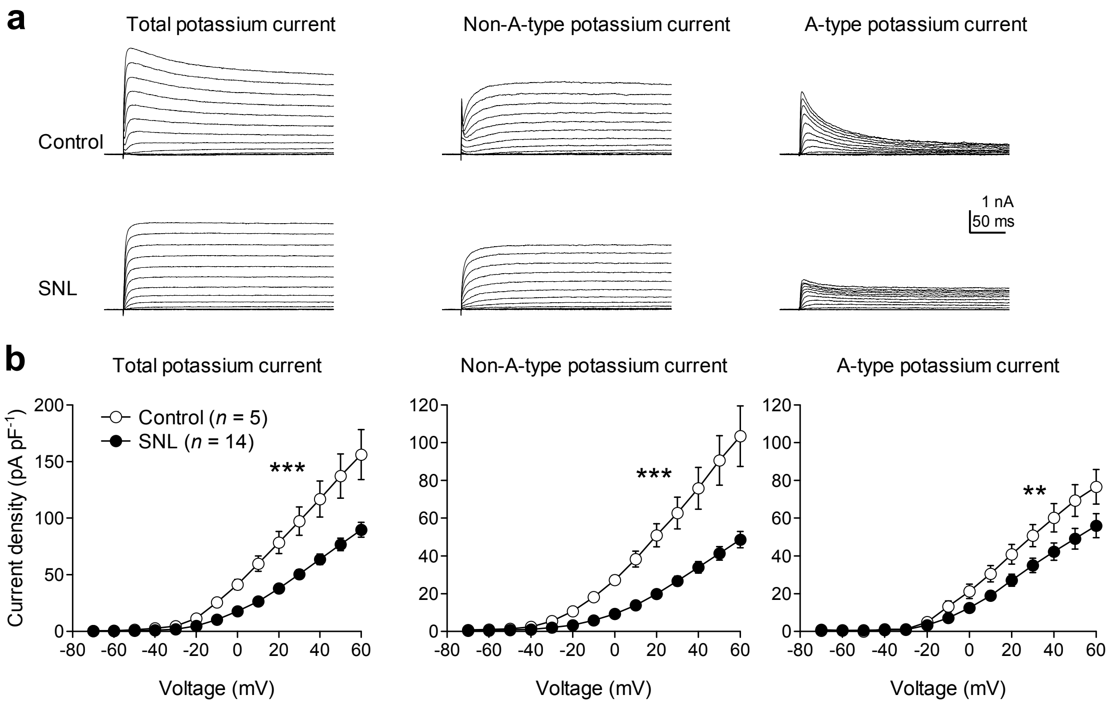


**Supplementary Figure 10 SNL diminishes potassium currents in DRG neurons.** Potassium currents were recorded in acutely-dissociated DRG neurons 7 days after SNL. (**a**) Representative traces of total, non-A-type and A-type potassium currents elicited by stepwise depolarization from -70 to 60 mV at holding potential of -80 mV in small DRG neurons. (**b**) Current density of each potassium current component plotted against voltage in small DRG neurons obtained from three rats. Error bars are s.e.m.. ***P* <0.01 and ****P* <0.001, two-way repeated-measures ANOVA.

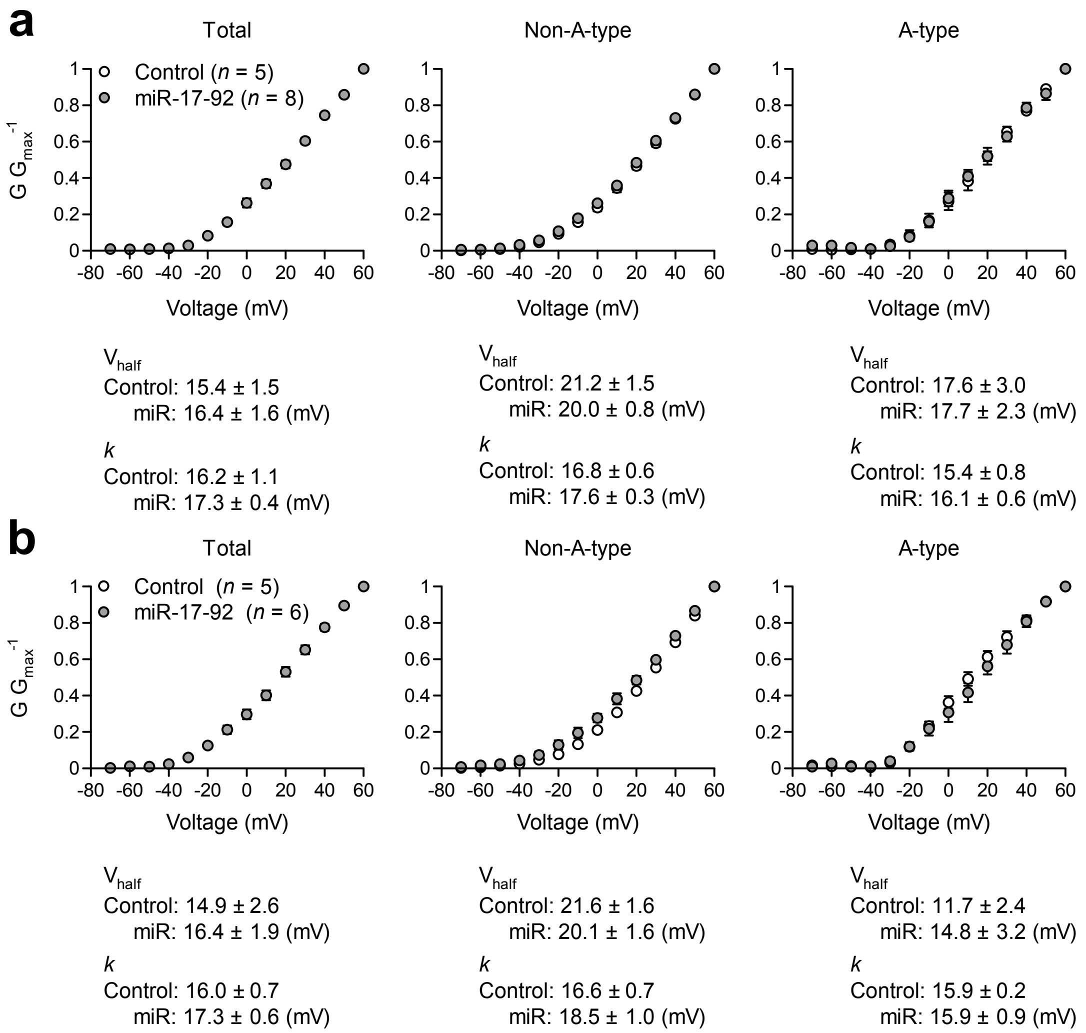


**Supplementary Figure 11 miR-17-92 does not affect the voltage-dependent activation of potassium channels.** Conductance-voltage curves of each potassium current component in small (**a**) and medium/large (**b**) DRG neurons obtained from three rats. Curves are described with the Boltzmann equation: $G\ G_{max}^{-1} = 1\ [1 + \exp(V_{half} - V_m\, k^{-1})]^{-1}$, where $V_{half}$ is the membrane potential at which 50% activation is observed, $k$ is the slope factor of the function and $V_m$ is the membrane potential. There was no significant difference in the values of $V_{half}$ or $k$ of each potassium current component. Error bars are s.e.m..

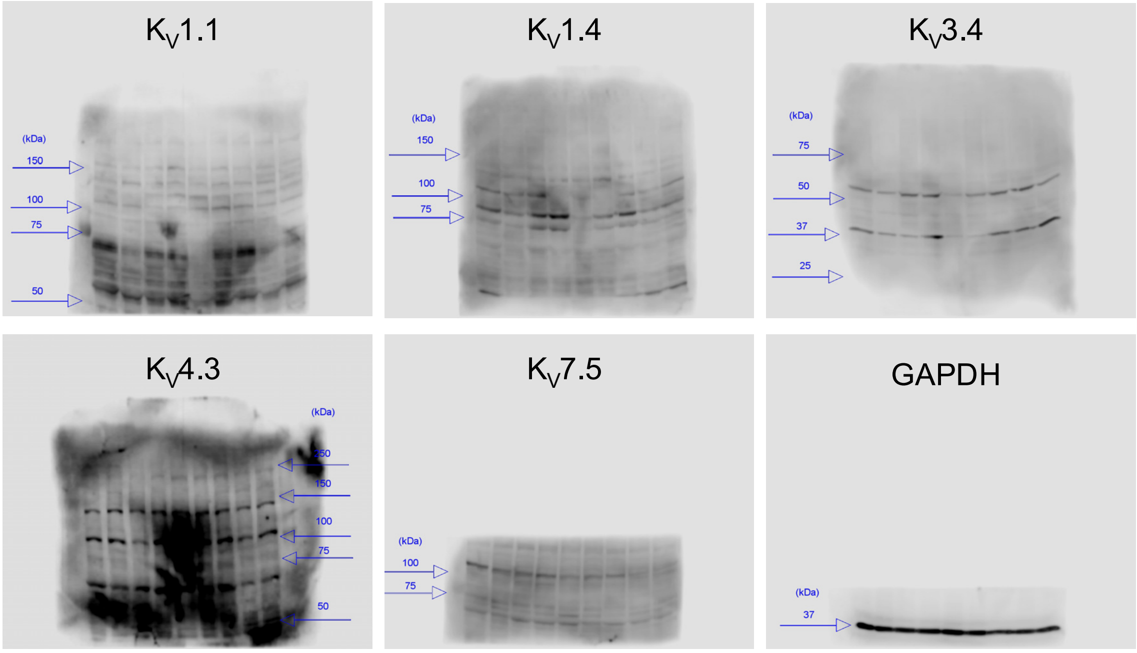


**Supplementary Figure 12 Uncropped scans of immunoblotting in the Figure 7.**

**Supplementary Table 1 Potassium channels that show significant expression changes with fold change of >1.5.**

| Gene Symbol | p | FC | Description |
|---|---|---|---|
| *Dpp10* | 0.0290319 | -2.6411946 | Rattus norvegicus dipeptidylpeptidase 10 (Dpp10), mRNA [NM_001012205] |
| *Dpp6* | 0.00858005 | -1.8871658 | Rattus norvegicus dipeptidylpeptidase 6 (Dpp6), mRNA [NM_022850] |
| *Kcna1* | 0.03943499 | -1.6309484 | Rattus norvegicus potassium voltage-gated channel, shaker-related subfamily, member 1 (Kcna1), mRNA [NM_173095] |
| *Kcna4* | 0.00736898 | -1.8086401 | Rattus norvegicus potassium voltage-gated channel, shaker-related subfamily, member 4 (Kcna4), mRNA [NM_012971] |
| *Kcnc4* | 0.02231177 | -1.562064 | Rattus norvegicus potassium voltage gated channel, Shaw-related subfamily, member 4 (Kcnc4), mRNA [NM_001122776] |
| *Kcnd2* | 0.00147561 | -2.1828494 | Rattus norvegicus potassium voltage-gated channel, Shal-related subfamily, member 2 (Kcnd2), mRNA [NM_031730] |
| *Kcnd3* | 0.00721676 | -2.817565 | Rattus norvegicus potassium voltage-gated channel, Shal-related subfamily, member 3 (Kcnd3), transcript variant 1, mRNA [NM_001270962] |
| *Kcng1* | 0.01335851 | -1.6778827 | Rattus norvegicus potassium voltage-gated channel, subfamily G, member 1 (Kcng1), mRNA [NM_001106545] |
| *Kcng4* | 0.00219923 | 1.8721493 | Rattus norvegicus potassium voltage-gated channel, subfamily G, member 4 (Kcng4), mRNA [NM_001107435] |
| *Kcnh2* | 0.04901159 | -1.6601135 | Rattus norvegicus potassium voltage-gated channel, subfamily H (eag-related), member 2 (Kcnh2), mRNA [NM_053949] |
| *Kcnh6* | 0.0131512 | -1.7392979 | Rattus norvegicus potassium voltage-gated channel, subfamily H (eag-related), member 6 (Kcnh6), mRNA [NM_053937] |

| | | | |
|---|---|---|---|
| *Kcnip1* | 0.03535908 | -2.159263 | Rattus norvegicus Kv channel-interacting protein 1 (Kcnip1), transcript variant 1, mRNA [NM_001261387] |
| *Kcnip4* | 0.0408274 | -1.8060249 | Rattus norvegicus Kv channel interacting protein 4 (Kcnip4), mRNA [NM_181365] |
| *Kcnj1* | 0.0339524 | -1.7434906 | Rattus norvegicus (clone ROMK2) epithelial K+ channel mRNA, complete cds. [L29403] |
| *Kcnj13* | 0.03243448 | 1.8928394 | Rattus norvegicus potassium inwardly-rectifying channel, subfamily J, member 13 (Kcnj13), mRNA [NM_053608] |
| *Kcnj3* | 0.02673408 | -2.6277072 | potassium inwardly-rectifying channel, subfamily J, member 3 (Kcnj3), mRNA [Source:RefSeq mRNA;Acc:NM_031610] [ENSRNOT00000007335] |
| *Kcnk16* | 0.04991378 | 1.55255 | Rattus norvegicus potassium channel, subfamily K, member 16 (Kcnk16), mRNA [NM_001109520] |
| *Kcnk4* | 0.01003591 | -2.4150057 | Rattus norvegicus potassium channel, subfamily K, member 4 (Kcnk4), mRNA [NM_053804] |
| *Kcnq5* | 0.00127116 | -1.8728338 | Rattus norvegicus potassium voltage-gated channel, KQT-like subfamily, member 5 (Kcnq5), mRNA [NM_001134643] |
| *Kcns1* | 0.0142297 | -1.8244975 | Rattus norvegicus potassium voltage-gated channel, delayed-rectifier, subfamily S, member 1 (Kcns1), mRNA [NM_053954] |
| *Kcnt2* | 0.03140713 | -1.7841375 | Rattus norvegicus potassium channel, subfamily T, member 2 (Kcnt2), mRNA [NM_198762] |
| *Scn1b* | 0.04527202 | -1.5205897 | Rattus norvegicus sodium channel, voltage-gated, type I, beta subunit (Scn1b), transcript variant 2, mRNA [NM_017288] |

**Supplementary Table 2 List of Assay ID of qPCR kit.**

| Gene | Assay ID (Life Technologies) |
|---|---|
| *Dpp10* | Rn01408809_m1 |
| *Gapdh* | Rn99999916_s1 |
| *Kcna1* | Rn00597355_s1 |
| *Kcna2* | Rn02769834_s1 |
| *Kcna4* | Rn02532059_s1 |
| *Kcnc4* | Rn01748431_m1 |
| *Kcnd3* | Rn04339183_m1 |
| *Kcnj8* | Rn01492857_m1 |
| *Kcnma1* | Rn00582881_m1 |
| *Kcnq5* | Rn01512013_m1 |
| miR-17 | 002308 |
| miR-18a | 000394 |
| miR-19a | 000395 |
| miR-19b | 000396 |
| miR-20a | 000580 |
| miR-92a | 000430 |
| Pri-miR-17-92 | Rn04229256_pri |
| Pri-miR-17-363 | Rn03465594_pri |
| *Scn1b* | Rn00441210_m1 |

**Supplementary Table 3 List of primer sequences.**

| Gene | Foward primer | Reverse primer |
|---|---|---|
| miR-17-92 | ggaattcggaacttctgcctattggct | ggaattcgtcagtgttcccatactcc |
| miR-17 | ggaattcggaacttctgcctattggct | ggaattcgcgccagcaggccctcccca |
| miR-18a | ggaattcagctgcctcgagaggggctg | ggaattcaaacagaactattgagacccc |
| miR-19a | ggaattctgccctaagtgctccttctg | ggaattcgcactataagcactttagtgc |
| miR-19b | ggaattctaagctccagcctcggcccgt | ggaattcgggtttgattttcccttacg |
| miR-20a | ggaattctatttacttcaagtgttgttg | ggaattcctcagtacggacagtcggac |
| miR-92a | ggaattcaaaagtctgtgaagacgtaaggg | ggaattcgtcagtgttcccatactcc |
| *Kcna1* 3'-UTR | gactagtaaaaagcaccaggcaagcaa | gactagtgaaggagtgcacttttggtg |
| *Kcna4* 3'-UTR | gactagtatctctttccccacctgccg | gactagtgaattctttctgtgcatgca |
| *Kcnc4* 3'-UTR | gactagtacacagagacctgccaagac | gactagtgcagatgctcacagaggagc |
| *Kcnd3* 3'-UTR | cccaagcttaaacactggacagagggcct | agagaactcagcagtaactg |
| *Kcnq5* 3'-UTR | gactagtgttctttgtttcctttctaa | gactagtagaggttgaccatttacaga |
| *Dpp10* 3'-UTR | cccaagctttggactacacttatacagag | cccaagctttgcatatgcacacgtgcgtg |
| *Scn1b* 3'-UTR | gactagttccgcctcaaggaagagcca | gactagttcgagtccaagaatctttat |
